# Implementing life cycle sustainability assessment for improved space mission design

**DOI:** 10.1002/ieam.4722

**Published:** 2023-01-18

**Authors:** Andrew R. Wilson, Massimiliano Vasile, Christie Maddock, Keith Baker

**Affiliations:** ^1^ Department of Mechanical & Aerospace Engineering, Aerospace Centre of Excellence University of Strathclyde Glasgow UK; ^2^ School of Engineering & Built Environment, Built Environment Asset Management (BEAM) Centre Glasgow Caledonian University Glasgow UK

**Keywords:** Life Cycle Sustainability Assessment, product development, space systems, systems engineering

## Abstract

Within the space sector, the application of Environmental Life Cycle Assessment (E‐LCA) is beginning to emerge as a credible and compelling method for scientifically quantifying environmental impacts of space missions. However, E‐LCA does not fully align with the concept of triple‐bottom‐line sustainability, while the combination of all three sustainability dimensions (environment, society, and economy) within a single life cycle study has thus far never been attempted within the space industry. Moving toward a Life Cycle Sustainability Assessment (LCSA) is, therefore, a logical next step for the space sector to allow these three sustainability dimensions to be addressed. Consequently, this article presents the underlying principles of a new LCSA framework for space missions and demonstrates its applicability for improving system‐level design concepts based on the interaction between sustainability dimensions. The framework was formed based on a systematic literature review to analyze the background, issues, and knowledge gaps related to life cycle methodologies, as well as context‐specific sustainability aspects. The framework has been implemented within a life cycle database called the Strathclyde Space Systems Database (SSSD). Using the SSSD, the framework was tested on a mission concept called Moon Ice Observation Satellite to demonstrate how changes in the design for a circular economy and other sustainability‐based principles will affect the functionality of the mission at the system level. It is envisaged that this framework will enable engineers to create sustainable space systems, technologies, and products that are not only cost‐efficient, eco‐efficient, and socially responsible, but also ones that can easily justify and evidence their sustainability. *Integr Environ Assess Manag* 2023;19:1002–1022. © 2022 The Authors. *Integrated Environmental Assessment and Management* published by Wiley Periodicals LLC on behalf of Society of Environmental Toxicology & Chemistry (SETAC).

## Key Points

- A framework for performing Life Cycle Sustainability Assessment studies of space systems has been proposed.
- The framework proposes best practice to improve system‐level design space concepts based on the interaction between sustainability dimensions (environment, society, economy).
- The application of the framework is demonstrated through a case study, which highlights how sustainable design measures can improve the profile of a space mission.
- Engineers can use this framework to develop sustainable space missions that can evidence their cost‐efficiency, eco‐efficiency, and social responsibility.

## INTRODUCTION

Outer space is a key resource in the pursuit of sustainable development. In this regard, the orbital environment around earth enables space missions to provide vital information that allows decision‐makers to measure progress and set plans of action on a variety of environmental, societal, and economic issues. However, despite their practical application, the adverse impacts caused by space missions to the earth's environment over their entire lifetime represent an aspect that is regularly overlooked, despite increasing calls for space environmentalism (Lawrence et al., 2022; Miraux, 2022; Palmroth et al., 2021; Wilson et al., 2022).

To address this, the European Space Agency (ESA) Clean Space Office has been pioneering the application of Environmental Life Cycle Assessment (E‐LCA) within the space sector to scientifically quantify and reduce geocentric environmental impacts of space missions since 2009 (European Space Agency, 2009). E‐LCA is a technique used to assess the environmental impacts of products, processes, or services over their entire life cycle. This technique is particularly useful in early mission design phases since around 80% of a product's environmental impacts are set by early design choices (European Commission, 2022a). Adverse impacts are more difficult to address the further into the design process that they are identified since several key decisions and constraints will have already been put in place, restricting the possibility for design modification (Chanoine et al., 2014; Sheldrick & Rahimifard, 2013).

Nevertheless, the use of E‐LCA does not fully align with the concept of sustainability envisioned within the 2030 Agenda for Sustainable Development, which seeks to “balance the three dimensions of sustainable development: the economic, social and environmental” (A/RES/70/1). Despite this, the possibility of combining all three sustainability dimensions within a single life cycle study within the space industry has only briefly been suggested by some researchers (Durrieu & Nelson, 2013; Harris & Landis, 2019; Maury et al., 2017; Viikari, 2004), with just one previous project having been initiated to investigate this further (Wilson, 2019). This is irrespective of the fact that the 2030 Agenda for Sustainable Development (A/RES/70/1), Guidelines for the Long‐term Sustainability of Outer Space Activities (A/AC.105/2018/CRP.20), and the European Union's Green Deal (Fetting, 2020) all increase the motivation and necessity for addressing the full spectrum of sustainability aspects within future space missions and technologies.

To address this, Life Cycle Sustainability Assessment (LCSA) could be used to enable the industry to introduce triple‐bottom‐line (TBL) considerations into the space mission design process. LCSA refers to the combination of E‐LCA, Social Life Cycle Assessment (S‐LCA), and Life Cycle Costing (LCC) into a single framework (Klöpffer, 2008). S‐LCA is an assessment type that can be used to predict the life cycle social and sociological aspects of products. LCC is an economic assessment that can be used to determine the entire cost of a product, process, or service over its entire life cycle including both one‐time and recurring costs. Therefore, rather than a model itself, LCSA is a framework of models designed to provide product‐related information in the context of sustainability and allow integrated decision‐making based on a life cycle perspective (Guinée, 2016).

The first and only attempt to apply LCSA to space missions is outlined in Wilson, 2019. However, the specificities of the space sector mean that applying such an approach is not a straightforward endeavor (Wilson, 2022). For this reason, defining robust methodological guidance in the form of a space LCSA framework is required to tailor this technique to the context of space mission design. Such an approach may, therefore, promote industrial stakeholders to become fully transparent in their operations by allowing them to scientifically quantify the overall sustainability performance of space missions and mitigate any potentially significant impacts (or hotspots) before they occur.

As such, the aim of this article is to feed LCSA into the decision‐making process of space missions to drive change at the system level while helping to create a truly sustainable space sector. To do this, a new framework will be presented that provides methodological guidance concerning the application of LCSA within the design process of early space mission design concepts. The framework has been developed to comply with several guiding principles, including the ESA E‐LCA guidelines (ESA LCA Working Group, 2016), the United Nations Environment Programme (UNEP)/Society of Environmental Toxicology & Chemistry (SETAC) LCSA guidelines (Valdivia et al., 2011), and the ISO 14040:2006 & ISO 14044:2006 standards, among others. Consequently, this should provide a credible and compelling new method for streamlining decision‐making in a more systematic and coordinated fashion, with the concept of sustainable development at its core. Finally, the proposed framework was applied retrospectively as part of a Phase 0/A concurrent engineering study that took place at the University of Strathclyde (Wilson & Vasile, 2022). This practical demonstration has been used as a case study to evidence the applicability of the developed framework, with the results highlighting how environmental, social, and economic performance can collectively act as a decision parameter in the space mission design process for improved system performance.

## METHODOLOGY

To fulfill the aim of this study, the development of the space LCSA framework will principally be based upon a literature review. After this, the process for implementing the new framework will be described through a case study, demonstrating the appropriateness of its application for early space mission design concepts. The methods for completing each of these steps are outlined below.

## LITERATURE REVIEW

The initial part of the literature review is designed to establish the guiding principles that will govern the formation of the space LCSA framework. This was based on known documents, which were considered an authoritative or front‐running source for life cycle practices in general, life cycle practices as applied to space systems, and space mission design in general. These were uncovered through prior knowledge, using simple searches of the Scopus database, Google Scholar, and the British Standards Online Library (BSOL). The reference documents captured by this approach are reported in Table 1 and were selected to ensure that each sustainability dimension, including trade‐offs and technical space‐related considerations, was fully captured by the space LCSA framework.

**Table 1:** Selected reference documents that form the guiding principles for the proposed space LCSA framework

Article no.	Reference document	Sustainability pillar			Decision‐analysis for LCSA (trade‐offs)	Technical considerations (space)	Description and overview
		Environment (E‐LCA)	Society (S‐LCA)	Economy (LCC)			
1	A/RES/70/1^a^		x		x		The 2030 Agenda for Sustainable Development, comprising the 17 Sustainable Development Goals (SDGs) and 169 targets.
2	A/RES/71/313^a^		x		x		A global indicator framework comprising 232 indicators to facilitate the implementation of the 17 SDGs and 169 targets.
3	Benoît‐Norris et al. (2020)		x				A document that provides a technical framework for calculating the social life cycle impacts of products.
4	ECSS‐S‐ST‐00‐01C					x	An international standard controlling the definition of all common terms used in the ECSS Standards System.
5	ESA LCA Working Group (2016)	x				x	Guidelines outlining a methodology for adapting the ISO E‐LCA standards to be more appropriate to space.
6	European Commission (2018)	x					A document providing instructions on how to develop PEFCRs for calculating the environmental profile of products.
7	Goedkoop et al. (2020)		x				A handbook describing a methodology for assessing the positive and negative social impacts of products and services.
8	Hannouf and Assefa (2018)	x	x	x	x		A journal article that presents a decision‐analysis framework for LCSA.
9	IEC 60300‐3‐3:2017			x			An international standard establishing a general introduction to the concept of life cycle costing and covers all applications.
10	ISO 14040:2006	x					An international standard that describes the principles and framework for E‐LCA.
11	ISO 14044:2006	x					An international standard that specifies the requirements and provides guidelines for E‐LCA.
12	ISO 26000:2010		x				An international standard that provides guidance on the underlying principles of social responsibility.
13	Klöpffer (2008)	x	x	x	x		A journal article that presents the LCSA concept for the first time, including suggestions on how results should be presented.
14	NASA (2015)			x		x	A guidance document covering the cost‐estimating methodology as applied at NASA.
15	Traverso et al. (2021)		x				Additional guidance relating to Benoît‐Norris et al. (2020) by the provision of further information on stakeholder subcategories.
16	Valdivia et al. (2011)	x	x	x	x		A publication presenting guidance on how to use and combine stand‐alone life cycle techniques to start an overall LCSA.
17	Wertz and Larson (1999)			x		x	A book that provides a comprehensive overview and summary into the theory and practice of designing spacecraft elements.
18	Wilson (2019)	x	x	x	x	x	A PhD thesis presenting a pathway for transitioning space E‐LCA toward space LCSA, which forms the basis for this work.

Abbreviations: ECSS, European Cooperation for Space Standardization; E‐LCA, Environmental Life Cycle Assessment; LCC, Life Cycle Costing; LCSA, Life Cycle Sustainability Assessment; PEFCRs, Product Environment Footprint Category Rules; S‐LCA, Social Life Cycle Assessment.

^a^ Although these reference documents relate to all three sustainability dimensions, for the purposes of LCSA, they are considered relevant for S‐LCA and decision‐analysis segments only.

This was complemented by a more comprehensive literature to provide additional evidence to inform the development of the space LCA framework and aid in the formation of rules. This was achieved by searching for peer‐reviewed journals, conference papers, books, reports, and standards based on a wide range of key terms including “life cycle assessment,” “social life cycle assessment,” “life cycle costing,” “life cycle sustainability assessment,” “multicriteria decision analysis,” and “space mission design,” among others. Several sources were used for this purpose, including the Scopus database, Google Scholar, BSOL, the National Aeronautics and Space Administration (NASA) technical library's public search engine TechDoc, proceedings published on conference websites, and various elements collected within the space industry from different sources. The reference section for each collected article was also searched in order to find additional research. In this regard, dozens of reference documents were uncovered. However, on review, only those considered to be most pertinent to the development of the space LCSA framework or those most reflective of general practice are reported in Table 2.

**Table 2:** Selected reference documents that provide additional evidence and aid in the formation of rules for the proposed space LCSA framework

Article no.	Reference document	Overview and relevancy to the space LCSA framework
1	Benini et al. (2014)	A technical report providing recommendations for normalisation methods and data for the PEF approach.
2	Benoît et al. (2009)	A document that provides a technical framework for calculating the social life cycle impacts of products (now outdated).
3	Benoît‐Norris et al. (2013)	Additional guidance relating to Benoît et al. (2009) by the provision of further information on stakeholder subcategories (now outdated).
4	Ciroth (2009)	A journal article discussing the parameters that affect cost data quality.
5	Chanoine et al. (2015)	A conference paper discussing the benefits and challenges of developing tools and integrating sustainability into the design of space activities.
6	Curran et al. (2004)	A journal article that provides a literature review on the state of the art in engineering cost modeling as applied to aerospace.
7	Diaz‐Sarachaga et al. (2018)	A journal article that analyzes the suitability of applying the SDG Index for assessing the fulfillment of the 2030 Agenda.
8	ECSS‐M‐ST‐10 Rev.1	An international standard describing key elements of coherent and integrated project planning in space projects and applications.
9	Finkbeiner et al. (2010)	A journal article that explores the status of LCSA for products and processes and how to present results.
10	Gluch and Baumann (2004)	A journal article discussing the usefulness of LCC for environmental decision‐making.
11	Hunt and van Pelt (2004)	A conference paper discussing the similarities and differences between NASA and ESA cost‐estimating methods.
12	ISO/TR 14062:2002	An international standard that describes the concepts and practices relating to ecodesign (now withdrawn).
13	Kayrbekova et al. (2011)	A journal article discussing the applicability of ABC as an alternative to conventional LCC in engineering design.
14	Keller et al. (2014)	A journal article providing results from a review on techniques, approaches, models, and conceptual tools for space program cost estimating.
15	Maier et al. (2016)	A journal article outlining a methodological approach for LCSA of development cooperation projects based on the SDGs and life cycle thinking.
16	Mrozinski et al. (2020)	A conference paper demonstrating a new method for parametric cost modeling for CubeSats.
17	Maury (2019)	A PhD thesis that sought to integrate space debris as an impact category within space E‐LCA.
18	Pagotto et al. (2021)	A book chapter that develops a framework for analyzing sustainability impacts of the Australian food industry, based on a literature review.
19	Rebitzer and Seuring (2003)	A journal article providing an overview relating to the methodology and application of LCC.
20	Sala et al. (2015)	A technical report outlining a state of the art and challenges for supporting product policies through S‐LCA.
21	Sala et al. (2018)	A technical report providing recommendations for a weighting approach for the PEF approach.
22	Shishko (2004)	A conference paper that discusses some potential approaches that can improve rigor and repeatability in the analogy costing process.
23	Sureau et al. (2018)	A journal article that reviews the criteria and indicators proposed to assess social and socioeconomic impacts of products.
24	Swarr et al. (2011)	A journal article providing guidance on environmental LCC via a code of practice.
25	Valdivia et al. (2021)	A journal article that establishes principles for the increased application and use of LCSA.
26	Velasquez and Hester (2013)	A journal article that provides an overview on the wide variety of MCDA methods developed.
27	Watson et al. (2006)	A journal article describing different cost estimation approaches as they relate to the aerospace supply chain.
28	Wilson and Vasile (2022)	A journal article demonstrating the use of LCE within the concurrent engineering process of space missions.
29	Wilson et al. (2021)	A conference paper outlining the results of a scoping exercise designed to map the specificities of space E‐LCA to the PEF approach.
30	Wulf et al. (2018)	A journal article demonstrating the selection of LCSA indicators based on the SDGs through a case study.
31	Zampori et al. (2018)	A technical report providing guidance for interpreting the results of life cycle studies.

Abbreviations: ABC, activity‐based costing; ECSS, European Cooperation for Space Standardization; E‐LCA, Environmental Life Cycle Assessment; ESA, European Space Agency; LCC, Life Cycle Costing; LCSA, Life Cycle Sustainability Assessment; MCDA, multicriteria decision analysis; PEF, Product Environmental Footprint; SDG, Sustainable Development Goal; S‐LCA, Social Life Cycle Assessment.

Collectively, the reference documents outlined in Tables 1 and 2 were used only where considered appropriate during framework development, as reported in the following section on the Framework Development. Although these reference documents relate to all three sustainability dimensions, for the purposes of LCSA, they are considered relevant for S‐LCA and decision‐analysis segments only.

## CASE STUDY

The developed space LCSA framework described in this article has been applied through a new tool called the Strathclyde Space Systems Database (SSSD). The SSSD can be used to facilitate LCSA of early space mission concepts and has already been used by actors across three continents. The tool contains over 250 validated space‐specific Life Cycle Inventory (LCI) data sets and includes several integrated Life Cycle Impact Assessment (LCIA) methods. Further information on the development of the SSSD is outlined in Wilson (2019).

Therefore, to demonstrate the new space LCSA framework, a prospective LCSA was conducted on the Phase 0/A Moon Ice Observation Satellite (MÌOS) concept using the SSSD. This means that although the overall concept is still at an early stage of development, the space mission will be modeled to represent a future, more developed stage. The MÌOS concept was created within a concurrent engineering environment at the University of Strathclyde, although the LCSA took place retrospectively. Primary data were obtained based on data deposited to the engineering model used in the concurrent engineering study and mapped to relevant SSSD LCI data sets. Additional data were also collected for elements not covered in the engineering model using relevant calculation and/or extrapolation techniques, expert knowledge, and default values contained within the SSSD.

The case study is intended to test the practicality of the space LCSA framework for reaching more sustainable designs of early space mission design concepts. The success of this framework will be measured by its ability to measure the sustainability performance of the MÌOS mission and then generate improvement opportunities to reduce its overall footprint. For this reason, the case study will neither focus on the development of the SSSD nor on the LCIA results of the space mission per se, but rather how the framework was implemented.

While the focus of this case study is on LCSA, the framework may also be applied as part of the concurrent engineering process of space missions through Life Cycle Engineering (LCE). LCE is an engineering technique used to assess the environmental, social, economic, and technical impacts of products, processes, and services over their entire life cycle by finding a balance between societal needs, economic growth, and minimizing environmental impacts in product engineering. Evidently, this makes this approach particularly well suited to concurrent engineering sessions that apply a model‐based systems engineering approach. In such sessions, dedicated space LCSA tools (such as the SSSD) can be used to scientifically quantify the sustainability footprint of early space mission concepts as the design progresses, with the LCE expert advising subsystem experts on how to redesign elements that are particularly problematic to lower adverse impacts without compromising mission objectives and requirements. Therefore, the proposed principles of the newly defined space LCSA framework can be integrated into such tools to enable conformity. The process for this, including all necessary adaptations to the proposed space LCSA framework below, is outlined in Wilson and Vasile (2022), which is exemplified through three case studies on SmallSats using the SSSD.

### Framework development

The application of LCSA in the space sector can generally be broken down into two levels, each of which follows the European Cooperation for Space Standardization (ECSS) space system breakdown from ECSS‐S‐ST‐00‐01. The first level follows a functional view and addresses system‐level activities, such as space systems, including the ground and launch segments. The second level represents equipment, components, materials, or processes. While in practice it is common for redesign activities to be conducted at the equipment, component, and material levels, targeting specifically the space mission design process as the desired level of application therefore places the emphasis on system‐level assessments, which forms the basis for this framework.

The space LCSA framework has been designed for implementation from either an LCSA or an LCE perspective. In this regard, it has been divided into two parts according to the decision‐analysis framework presented by Hannouf and Assefa (2018). The first is the modeling of each life cycle technique to understand the total environmental, social, and economic impacts of space missions. The second focuses on its direct applications through a decision‐analysis technique called multicriteria decision analysis (MCDA), which can be used to handle the multifunctional aspects of each sustainability dimension and assessment type to enable a decision to be made. For this reason, the new space LCSA framework considers MCDA as a vital additional step within the decision‐making process.

**Figure 1:**
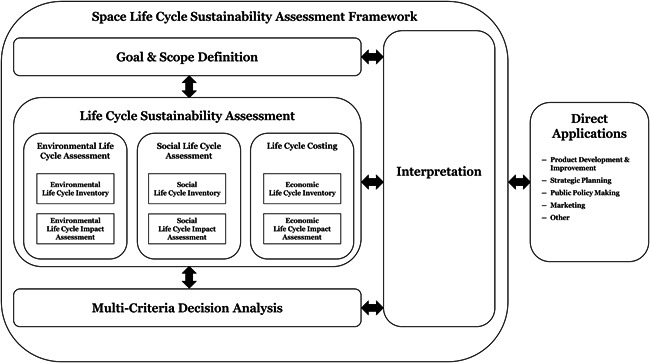
LCSA framework for the sustainable design of space missions (Wilson, 2019). LCSA, Life Cycle Sustainability Assessment

An overview of the new space LCSA framework is outlined in Figure 1 and was developed based on the reference documents outlined in Tables 1 and 2. As such, the new framework should be seen as an extension of these guiding principles rather than an alternative to them. This will be further explained throughout the remainder of this section.

### Goal and scope definition

The fulfillment of mission requirements and objectives as well as the role of the LCSA will ultimately inform the purpose of the goal and scope definition. Valdivia et al. (2011) strongly recommend the use of a common goal and scope definition when conducting an LCSA, taking into account the different requirements of the three assessment types, including the functional unit (FU) and system boundary. The FU is a quantified performance of a product system for use as a reference unit and is what all of the inventory inputs and outputs, as well as impact assessment results should be related. The system boundary specifies which unit processes are included as part of the product system.

**Figure 2:**
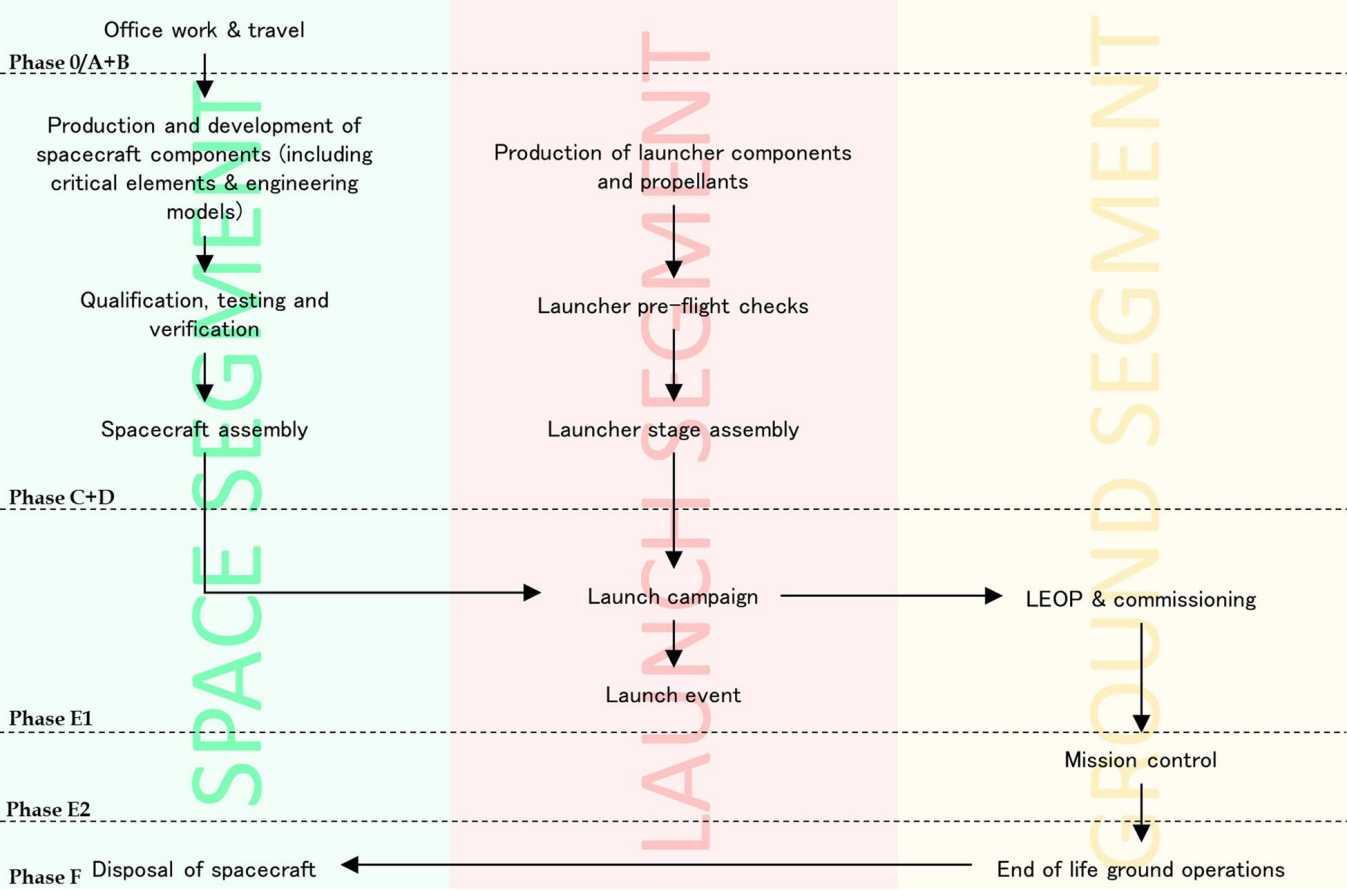
Generic system boundary of a typical space mission. *Source*: Adapted from ESA LCA Working Group (2016)

The ESA space E‐LCA guidelines, which are outlined further in the following subsection, provide excellent guidance on these aspects from an environmental perspective (ESA LCA Working Group, 2016). In this regard, as very few space missions serve an identical purpose or function, obtaining an FU that enables comparison based on the “function” of a satellite is very difficult. As such, the guidelines provide a common, simplified FU that is defined as “one space mission in fulfilment of its requirements.” Although this can be applied to multiple space missions, comparative assessments at the system level are not recommended by the ESA guidelines because of the varying requirements and specifications of space missions, even of the same mission class. Efforts to create dedicated FUs for different mission classes are currently being investigated in a scoping exercise (European Commission, 2022b), based on a recommendation made by Wilson et al. (2021). The suggested system boundary of the ESA space E‐LCA guidelines covers the sum of space segment, launcher segment, and ground segment across all phases (0/A to F) for both the payload and the platform of the space mission in accordance with ECSS‐M‐ST‐10 Rev.1. Infrastructure impacts may also be considered separately. A representative systems boundary diagram of a typical space mission is outlined in Figure 2.

However, careful attention should be paid to the system boundary, since each life cycle technique may have slightly different boundaries based on their relevancy to the overall assessment. Identical system boundaries should be applied to each of the three approaches whenever possible. Additionally, other small methodological differences can be considered within the goal and scope in order to determine how they might affect the study. For example, S‐LCA may require the selection of an activity variable to measure the share of a given activity as it relates to each unit process and LCC may consider the use of a work breakdown structure (WBS) that adopts a life cycle actor perspective (e.g., supplier, manufacturing, user, or consumer) to facilitate consistent data collection along the full life cycle. Furthermore, the scale of the relationship between the activity and unit process can massively impact the results and is therefore an important consideration within the goal and scope definition of an LCSA (Finkbeiner et al., 2010).

### Inventory analysis and impact assessment

The LCI analysis and LCIA of each assessment type should be based on a combined approach using the most relevant methodologies in the context of LCSA when it is applied to early space mission design phases. When combining the three assessment types into a single framework, it is common practice for S‐LCA and LCC to align with the principles and rules of E‐LCA. This places an added importance of defining methodological rules for space E‐LCA and tailoring both S‐LCA and LCC to be consistent with this in order to produce scientifically robust and sound analyses.

#### Environmental Life Cycle Assessment

Environmental Life Cycle Assessment is internationally standardized through the ISO 14040:2006 and ISO 14044:2006 standards and has been increasingly applied within the European space industry over the past decade to scientifically quantify environmental impacts of space missions over their entire life cycle. To help facilitate this, ESA released a consolidated set of guidelines in 2016 that act as primary guiding principles that should be applied when conducting a space E‐LCA. The guidelines tailor the methodological rules contained within the ISO 14040:2006 and ISO 14044:2006 standards to be more appropriate to the space sector without risking noncompliance. They are also oriented as closely as possible with the Product Environmental Footprint approach to better align with the strategic goals of the European Commission and are available to European stakeholders upon request. As such, E‐LCA shall be conducted in a manner that is consistent with the approach specified by these guidelines and its other associated adaptations and/or extensions such as Maury (2019) and Wilson et al. (2021).

In particular, the ESA guidelines cover all aspects of space E‐LCA, including the LCI and LCIA phases at the two levels defined by ECSS‐S‐ST‐00‐01, including primary and secondary data considerations, cut‐off criteria, environmental indicators (including space applicable factors), results communication, and more. It also provides a small number of simplistic LCI data sets that can be used in conjunction with dedicated space life cycle databases or software, such as the SSSD or ESA E‐LCA Database. However, given the rigorous guidance provided by this source, the focus of the remainder of this subsection will be on tailoring S‐LCA and LCC to align with E‐LCA.

#### Social Life Cycle Assessment

Increased levels of public perception on social responsibility in recent years have placed an added pressure on organizational transparency and the justification of public budget spending. For this reason, while space S‐LCA is still finding its feet, socioeconomic impact assessments (SEIAs) have been applied more widely within the space sector. These are systematic methods of analysis that are commonly applied to evaluate socioeconomic and cultural impacts of a proposed project or space mission. Comparatively, no space S‐LCA studies are known to have ever taken place, besides those listed in Wilson (2019) and Wilson and Vasile (2022).

This means that there is currently an absence of guidance on space S‐LCA guidance, despite a clear need for quantifying the social impacts of space systems to ensure the development of socially responsible mission concepts. A dedicated project is about to get underway at the University of Strathclyde, which is hoped to produce detailed preliminary guidance on the topic, expanding on the principles developed by Wilson (2019). However, in the meantime, these foundational principles can be derived as part of this space LCSA framework.

Based on this, it is suggested that the S‐LCA inventory is formed using mainly a burden‐based approach in order to be more comparable with E‐LCA and LCC, hence replicating their general methodologies. Although it can be contemplated that space missions may create a distinctly positive social impact (e.g., through environmental monitoring, catastrophe prevention, etc.), it can generally be considered that SEIAs are a more appropriate assessment type to capture such impacts. Regardless, due to the nature of S‐LCA, an added emphasis is placed on creating an evaluation scheme that can handle both positive and negative social impacts in a consistent way (as discussed later in this section). This leans toward a risk‐based approach (as the SOCA database) where positive impacts are represented by lower risk classes while neutral to negative impacts would have a higher risk class.

The LCI data can be collected across the six stakeholder categories and their associated subcategories as defined by Benoît‐Norris et al. (2020), at the country, organizational, or product level (Sureau et al., 2018). The applied perspective will depend on data availability and goal and scope definition. However, due to the unique nature of space systems and the fact that they are not commonly created within a mass production cycle, this disfavors a product‐level approach, leaving either a country‐level or organizational‐level approach. Under each subcategory, a range of indicators are provided by Traverso et al. (2021). Similarly, Wilson (2019) produced a list of 105 new social indicators in accordance with the ISO 26000:2010 standard and are tailored to the specificities of the space sector and its supply chain. However, these were based on now outdated guidance (Benoît et al., 2009; Benoît‐Norris et al., 2013) and need to be updated according to Benoît‐Norris et al. (2020) and Traverso et al. (2021). Additionally, in order to align with the 2030 Agenda for Sustainable Development, these indicators were developed based on the Sustainable Development Goals (SDGs), including their targets and indicators (A/RES/70/1; A/RES/71/313), in a similar manner to Maier et al. (2016) and Wulf et al. (2018). The indicators have been implemented within the SSSD and it is suggested that they are used as a basis for space S‐LCA until more detailed guidance is issued. It should also be noted that a new ISO standard is currently under development to define the principles and framework of the approach (ISO/AWI 14075).

Each of the 105 social indicators has been designed with an appropriate unit of measurement and evaluation scheme with relevant benchmarks on which to measure LCI results, similar to the method outlined by Goedkoop et al. (2020). This allows social impacts to be defined by levels of risk, ensuring comparability. The benchmarks should have set uniformed intervals on which numerical levels of risk can be determined. This is a particularly advantageous approach since S‐LCA results can be based on a mixture of qualitative and quantitative data. Making decisions based on qualitative data can lead to high levels of subjectivity into the analysis while basing decisions only on quantitative data could offer too narrow a view for proper decision‐making. By defining social aspects by levels of risk, all impacts (both qualitative or quantitative) can be measured based on these benchmarks and compared in a single unitary value (social risk). These data sets can then be combined with E‐LCA processes using the number of person‐hours accrued during the process under study as the activity variable. However, it is important that an external auditor validates the S‐LCA data sets, particularly where the level of risk assigned could have been done through a qualitative assertion (Sala et al., 2015).

In terms of LCIA methods, the stakeholder categories previously mentioned or the SDGs shall be used as impact categories. Since the applied scoring mechanism uses a single common unitary value (i.e., social risk), the impact categories can therefore be aggregated to form a single score for S‐LCA, thereby facilitating both options on which to evaluate LCSA results as proposed by Klöpffer (2008).

#### Life Cycle Costing

Life Cycle Costing is already a fundamental element in the early definition phases of space concepts since cost is generally considered to be a major driver in terms of mission viability. For this reason, LCC is perhaps the most mature of the three life cycle techniques within the context of aerospace, with the cost‐estimating process for early‐phase space mission concepts given in Wertz and Larson (1999). In this regard, costs can be broken down into three main phases: Research, Development, Test, and Evaluation (RDT&E); Production; and Operations and Maintenance (O&M). For each of these phases, a work breakdown structure (WBS) is defined, dividing costs among the basic elements of a space architecture, namely, the space, ground, and launch segments, as well as management and systems engineering (program‐level costs).

According to Swarr et al. (2011) and the IEC 60300‐3‐3:2017 standard, the LCI phase involves the collection and calculation of monetary data. Typically, dedicated cost‐estimating models will be used for this purpose. As outlined by NASA (2015), three cost‐estimating methods are mainly used during the space mission design process, with the choice of method dependent on concept maturity. The first is a parametric estimation approach, where simplistic statistical models, extrapolations, or cost‐estimating relationships (CERs) are created based on historical cost data, correlating the cost of an element to physical, technical, and performance parameters that are known to strongly influence costs. Such techniques are often used during conceptual studies in early mission design stages where design details are scarce or there is limited mission definition. The second is a top‐down analogy‐based estimating approach, where the cost of a similar item is used as a baseline and is then adjusted for differences in size, complexity, technology‐readiness levels (TRLs), and so forth. The baseline item(s) and scaling method used can be based on expert judgment or more formal methods (e.g., Mrozinski et al., 2020; Shishko, 2004). This technique is typically applied once a mission design is more adequately defined, but there are still insufficient actual cost data to use as a basis for a detailed approach. Lastly, there is the grassroots methodology, which is a bottom‐up estimate of every activity in the project's WBS, including overheads. It is applied when there is adequate project maturity, which allows far more detailed cost data to be accumulated despite being a lot slower and more labor‐intensive. Depending on the design details available, these methods can be used at the system, subsystem, or component level.

In terms of this framework, parametric and analogous cost estimation techniques could be considered as most appropriate for early space mission design concepts. However, NASA (2015) states that none of these techniques is individually sufficient to accurately estimate the life cycle cost of a space mission. For this reason, several different cost models and techniques often need to be used in conjunction for this purpose. In comparison to NASA, ESA applies a mixture of in‐house built CER tools based on Excel and commercially available cost estimation tools (Hunt & van Pelt, 2004).

Despite this, besides the three phases outlined by Wertz and Larson (1999), when applied as part of the space LCSA framework, there is a need to ensure complete coverage of system boundary. To achieve this, another interesting cost‐estimating methodology that could be incorporated is activity‐based costing (ABC). ABC is an approach whereby costs of organizational activities are identified and assigned to products, processes, services, and activities according to the actual consumption of each. According to Curran et al., the implementation of this technique is based on activity pools that are a collective set of activities (Curran et al., 2004). Each activity pool is then allocated to a specific cost driver as a base (i.e., the amount of an activity used). All overhead costs are then determined and calculated per cost driver. As such, the method assigns more indirect costs elements into direct costs compared to conventional costing approaches and evidently aligns closely with the E‐LCA modeling approach. Besides this, one of the main advantages of using this method is that the number of cost pools used to assemble overhead costs can be expanded. As such, new bases on which to assign costs are produced (i.e., FUs as cost drivers), which allows the nature of several indirect costs to be altered in a way that makes them more traceable to certain activities. Although this is a far more labor‐intensive approach, the method can often lead to a significantly more thorough and informative costing analysis (Keller et al., 2014).

Therefore, due to its applicability during conceptual studies, a parametric and/or analogous methodology is proposed for LCC using an ABC estimating approach. This is more in line with the life cycle methodology than the conventional LCSA approach and allows grouped LCI data sets to be generated using specialized life cycle modeling software that has been explicitly developed to handle life cycle activity‐related data of products (Watson et al., 2006). This is because the method allows other overhead elements that are not typically included within space systems cost engineering models (e.g., cost of heating and/or electricity consumption during design work) to be considered. As such, a more complete cost model can be developed that better aligns with the current LCC methodology while covering the sum of space, launch, and ground segment for the system boundary under study. As such, the main difference between the LCC approach proposed by this framework and cost analyses that typically occur during space mission design studies is the complete number of cost pools used to assemble overhead costs, which leads to a more detailed assessment being conducted. Additionally, when applied within concurrent engineering, this approach can also be seen to be more resource efficient since it requires just one discipline expert to cover three assessments and eliminates the need for a separate cost expert (Kayrbekova et al., 2011).

However, appropriate aggregation of costing data is extremely important in cases where co‐products are produced. This is because some expenses, particularly overheads, cannot always be directly related to a product (Kayrbekova et al., 2011). Additionally, since costs might occur for different actors, it is also important to differentiate and select which costs and cost bearers are included within the assessment (Gluch & Baumann, 2004). For this reason, caution must be exercised when calculating the LCI or using cost models. According to Ciroth, another important consideration during this phase involves the selection of an appropriate discount rate (Ciroth, 2009). Discount rates are used to convert future costs associated with a product system into a net present value, thus accounting for future inflation rates. Cost data might also be gathered in different currencies over different time periods (Swarr et al., 2011). LCI data will therefore need to refer to a common currency at present value using appropriate exchange and discount rates.

Lastly, like S‐LCA, while the LCIA phase is not strictly required within LCC, it has been considered as a mandatory requirement within this framework to make the results comparable to those of E‐LCA and S‐LCA. As such, all monetary values should be aggregated into economic cost categories, life cycle stages, activity types, or cost elements. Since this methodology uses a single unitary value, it is also recommended that a single score is generated and integrated as an impact category within E‐LCA for the same reasons as those stated for S‐LCA (Rebitzer & Seuring, 2003).

### Multicriteria decision analysis

Before the significance of the LCIA results can be interpreted, it is important that decision‐makers are able to understand the severity and trade‐offs between sustainability dimensions. As such, a systematic and structured decision‐analysis technique is required to assist decision‐makers to evaluate and improve the sustainability performance of a product. However, the plethora of impact categories across the three different assessment types creates difficulties for decision‐makers in terms of their ability to handle such plentiful and diverse forms of data (Valdivia et al., 2021). Three options are generally available for presenting results and making decisions:

- Option 1: Results of each assessment are presented separately using their own impact categories.
- Option 2: Results of S‐LCA and LCC are added as impact categories within E‐LCA.
- Option 3: Results are presented as a single sustainability score.

Option one is perhaps the most scientific and provides most depth but can be overwhelming due to the amount of impact categories. Option two is generally more advised to simplify decision‐making, particularly if the S‐LCA and LCC impact categories have been developed using common units. Despite this, when applying E‐LCA within concurrent design, ESA found that the high number of impact categories included within the assessment significantly complicated decision‐making due to the iterative nature of the process (Chanoine et al., 2015). Additionally, neither of the first two options adequately addresses the interaction between the sustainability dimensions or the burden‐shifting effect that occurs during system redesign, which implies an obvious risk of cherry‐picking and suboptimized decision‐making.

For this reason, option three presents a more credible decision‐analysis technique to enable trade‐offs between sustainability dimensions. Despite this, it is considered only to be appropriate for the identification of hotspots and not for results presentation (with the exception of within concurrent engineering studies because the technique simplifies the decision‐making process and reduces the learning curve of engineers who may have limited time within such studies).

In relation to option three, a commonly used technique is MCDA, which is frequently applied within decision‐making to address problems with conflicting goals, handle diverse forms of data, and reach conclusions, particularly when there could be multiple perspectives as with sustainability issues. It is increasingly being applied to LCSA studies to address the multidimensional results of LCSA and is recognized by many researchers as a critical component of LCSA. As documented by Velasquez and Hester (2013), various methodological approaches exist for MCDA, but of particular relevance to LCSA is the multiattribute value theory (MAVT) approach. This quantitatively compares a set of attributes or criteria by calculating their performance with respect to a given objective. In this respect, the MAVT approach can be used to assign real numbers to different alternatives in order to produce a preference order on the alternatives consistent with decision‐maker value judgments. The technique is particularly useful when assessing trade‐offs between conflicting criteria and combining dissimilar measurement units. The MAVT approach is typically based on the following weighted sum formula:

(1) $$v\left(a\right)=\sum_{i=1}^{I}\mathrm{wi}\;\mathrm{vi}\left(a\right),$$

where *v(a)* is the overall score of product *a* on sustainability dimension *v, wi* is the weighting factor for impact category *i, vi(a)* is the score reflecting the performance of product *a* on impact category *i*, and *I* is the total number of impact categories.

In this regard, a score reflecting performance for each impact category should be calculated through the sum of normalization and weighting procedures when applied to LCIA results. Normalization is the magnitude of impact relative to reference information, while weighting expresses the relationship between normalized impacts and politically determined goals or targets. To align with the approach adopted within the ESA E‐LCA guidelines, the recommended method for E‐LCA is to apply normalization and weighting values for E‐LCA developed by the European Commission (Benini et al., 2014; Sala et al., 2018), while new factors for S‐LCA and LCC as single score impact categories are outlined in Wilson and Vasile (2022).

Supplementary weighting factors are also suggested by Wilson and Vasile (2022) for each impact category with respect to its parent assessment type to reflect their respective sustainability dimension. This is used to determine the relative contribution of each impact category in terms of a single score. In this case, the most dominant political framework for sustainability currently in existence can be used to reflect this. This allows the three dimensions of sustainability to be appropriately balanced according to the level of concern given to each with respect to the contents of 2030 Agenda. In this respect, Diaz‐Sarachaga et al. (2018) group the 17 SDGs and their associated 169 targets into environmental, social, economic, and governance categories, and use the Delphi methodology to highlight the percentage of goals and/or targets dedicated to each sustainability dimension. Based on this study, it was found that the weighting factor for the environment was 18% in comparison to 53% for social and/or governance and 29% for the economy. Equation (1) can then be used again, based on *v(a)* for each assessment type and the above weighting factors to obtain an overall single sustainability score.

Lastly, it should be noted that although normalization and weighting procedures are listed as optional elements according to the ISO 14040:2006 and ISO 14044:2006 standards, their use in MCDA is considered vital to the space LCSA framework, with a distinct overlap in relation to the interpretation phase.

### Interpretation

In terms of interpretation, the ESA E‐LCA guidelines state that environmental hotspots should be identified during this phase (ESA LCA Working Group, 2016). This is a critical element of the decision‐analysis process, with common techniques to determine hotspots including contribution analyses, dominance analyses, influence analyses, or anomaly analyses (Zampori et al., 2018). However, in accordance with this framework, MCDA is considered as the foundation for hotspot analysis. Building upon the LCSA decision‐analysis framework proposed by Hannouf and Assefa (2018), it has been considered that the interpretation phase within this framework should consist of the following steps (which should be repeated with each iteration of analysis):

- Hotspot identification: Informed by the relative contribution to the MCSA single score result.
- Objective identification: A set of objectives are proposed to address the defined hotspots.
- Solution generation: A range of possible solutions should be sought in line with these objectives.
- Solution evaluation: All identified solutions are analyzed in order to determine their effectiveness.
- Trade‐off analysis: Trade‐offs are evaluated collectively for all solutions to determine which delivers the most optimal sustainability performance in relation to the sustainability dimensions and technical requirements.
- Implementation and/or recommendations: The selected solution can then be recommended or implemented within the system design model.

Although the process is the same, it is important to consider the potential of the above steps for generating solutions depending on the study application (LCSA vs. LCE). Once a hotspot is defined in an LCSA study, it can be investigated further at the subsystem level. An example of this is presented in Wilson (2019) for a battery module. In this analysis, when using MCDA to determine hotspots, it was found that social aspects and costs were the most dominant sustainability dimensions. It went on to show the respective trade‐offs concerning the implementation of potential improvement measures, exemplified by addressing the social hotspot of health and safety by looking into methods for introducing more stringent health and safety training for employees and the net impact across all sustainability dimensions. In comparison, when addressing hotspots during concurrent engineering sessions through LCE, only system‐level improvements are possible due to time constraints. This means that either reducing the quantity of the hotspot or replacing and/or phasing out the hotspot is entirely possible in real time. This is discussed further in Wilson and Vasile (2022), which also provides additional guidance on the adaptation of this framework to facilitate LCE within concurrent engineering sessions of space missions.

The interpretation phase should also seek to provide a set conclusions, limitations, and recommendations while addressing uncertainties and data quality where possible. However, uncertainty and data quality are aspects that are currently lacking in space LCA. This is seen by many as being a priority issue that needs to be addressed in the near future. Although critical reviews are required for E‐LCA in the case of a comparison, according to (the now outdated) ISO/TR 14062:2002, they are not an essential component of ecodesign. If these standards are to be followed for LCSA, this also means that a critical review is not strictly required for LCE but is perhaps advisable. This could be conducted by independent experts between space mission design sessions or when a final design is reached, particularly if the results are intended for public disclosure.

### Direct applications

Another important consideration of this framework is how the outcomes of space LCSA studies will be used. This places a need for the findings to be passed to the relevant decision‐makers (Valdivia et al., 2021). As outlined in the ISO 14040:2006 and ISO 14044:2006, numerous direct applications for life cycle studies exist. However, from the early application of E‐LCA within the space sector, perhaps the most common is for engineers to receive the input from the LCSA expert to update and improve the design through reiteration. This can either be initiated between design sessions through LCSA, or by applying this framework within the concurrent engineering process by adapting it toward LCE, as specified by Wilson and Vasile (2022). Regardless, according to Wilson (2019), the multitude of possible applications of the LCSA approach for space systems makes it a powerful tool for the space industry to:

- comply with current and future legislation,
- cut costs,
- facilitate technological development and advance with the times,
- respond to consumer demand for sustainable products (creating a competitive advantage), and
- create a more sustainable space sector.

## FRAMEWORK IMPLEMENTATION

The applicability of this framework will be tested on a Phase 0/A SmallSat concept called MÌOS, which was designed at the University of Strathclyde in September 2017 as part of the ESA Academy's First Concurrent Engineering Challenge. MÌOS stands for Moon Ice Observation Satellite, which is a derivation of the Scottish Gaelic word for month, and less commonly, moon. The mission has an aim of collecting data on the lunar micrometeorite and radiation environment as well as detecting the presence of water and ice content on the lunar South Pole in view of a future moon base.

**Figure 3:**
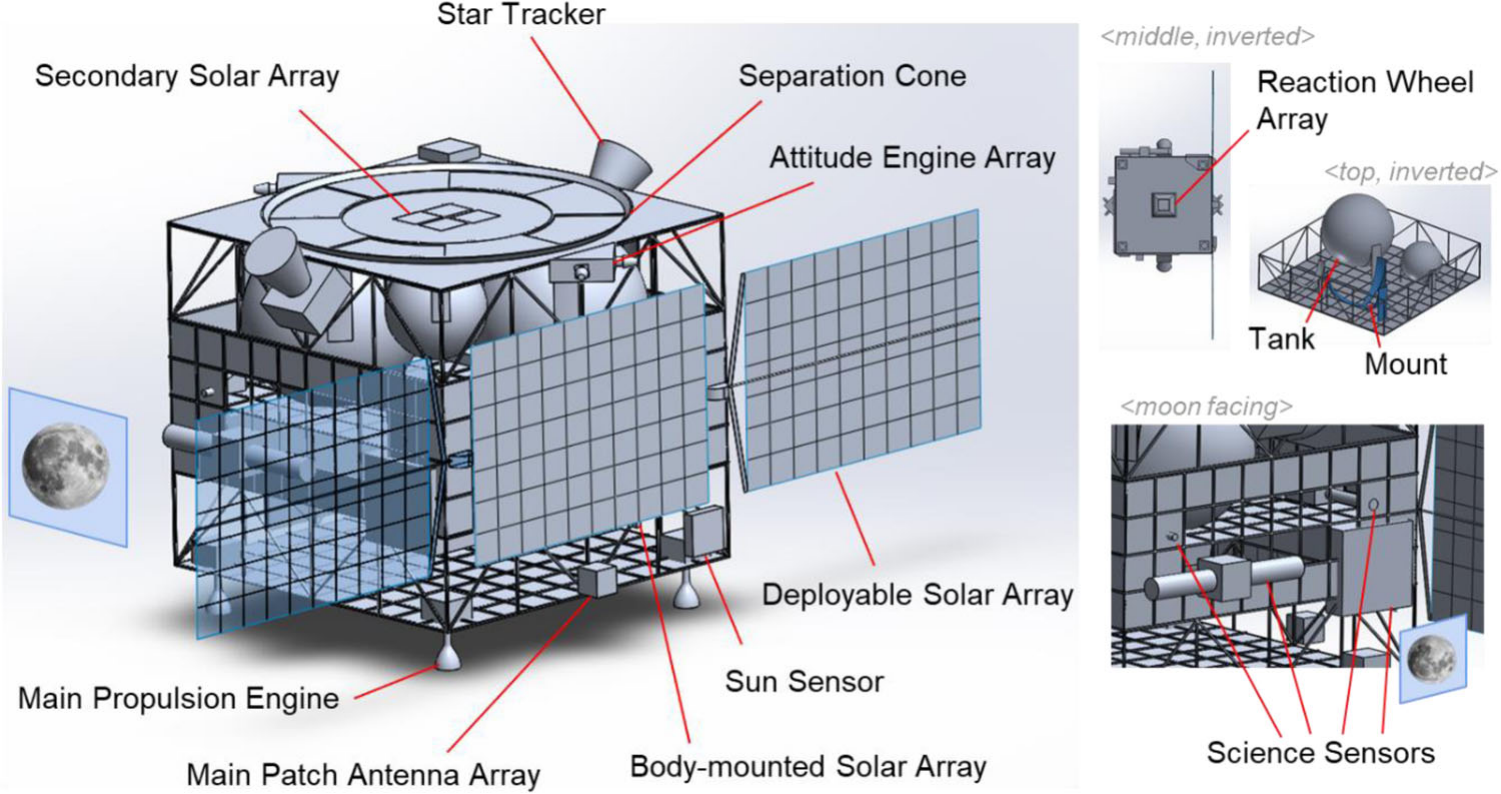
Configuration of the MÌOS (Moon Ice Observation Satellite) space mission design concept

The mission objectives and requirements that drove the design are outlined in Table 3. The final baseline design had a total wet mass of 286.04 kg including mass margins and the launch adapter. According to the mission requirements, this was 13.96 kg within budget. The total mission duration is 913 days, consisting of a single satellite in a frozen lunar orbit with a maximum eclipse time of 160 minutes. It is sun pointing for most of the lunar orbit, with a minimum altitude of 82 km and a maximum altitude of 119 km at the Lunar South Pole. The mission concept uses a narrow‐angled camera for taking pictures of the water and/or ice content and a wide‐angled camera for the radiation and/or micrometeorite environment. The configuration of the components can be seen in Figure 3. At the final design review, it was concluded that MÌOS was a solid and sound design concept that satisfied all mission requirements with no major design flaws.

**Table 3:** Mission objectives and requirements for the MÌOS space mission design concept

MIS‐OBJ‐01	The mission shall make pictures of South pole areas with high expected water and/or ice content, with a resolution of 10 m/pixel.
MIS‐OBJ‐02	The mission shall observe the lunar radiation and micrometeorite environment.
MIS‐OBJ‐03	The mission shall observe the water and/or ice content of the lunar South Pole.
MIS‐R‐01	The mission shall consist of a single satellite or a single plane constellation.
MIS‐R‐02	The mission shall stay in lunar orbit for two years.
MIS‐R‐03	The mission shall be launched using an Ariane shared GTO.
MIS‐R‐04	The mission shall be compatible with any launch date.
MIS‐R‐05	The total combined mass of the whole system shall be 300 kg.
MIS‐R‐06	The mission should use COTS components.
MIS‐R‐07	The mission shall have an end‐of‐life disposal maneuver.
MIS‐R‐08	The mission shall use direct to earth communication.
MIS‐R‐09	Applicable documents: CDF margin philosophy.

Abbreviations: CDF, Concurrent Design Facility; COTS, Commercial Off‐The‐Shelf; GTO, Geostationary Transfer Orbit; MÌOS, Moon Ice Observation Satellite.

Therefore, to demonstrate the applicability of the LCSA framework, a post‐Concurrent Design Facility LCSA study will be performed on this design concept. As part of this case study, the life cycle sustainability impacts of the MÌOS baseline design will first be investigated and then compared with an adaptation of the same model where two predetermined “sustainable design” options will be implemented. The first of these options targets the most substantial hotspot identified within MÌOS baseline design according to the most greatly impacted sustainability dimension according to MCDA. The second was to investigate the possibility of replacing the propellant with a high‐performance green propellant (HPGP) to test if there is a case for this switch within future design sessions of the MÌOS mission. The collective influence of these options on LCIA results will be investigated at the system level.

### Baseline design

The goal of this study was to inform decision‐makers of the most prominent sustainability impacts of the MÌOS concept before any further iterations and/or design sessions occur. The FU was set as “the MÌOS mission in fulfilment of its objectives and requirements.” The system boundary covers the sum of the space, ground, and launch segment across all phases of the space mission, consistent with the system boundary diagram found in Figure 2. However, it should be noted that there are no environmental impacts stemming from the deorbiting maneuver (besides ground station work) as the MÌOS mission was designed to crash into the moon at the end of its life; hence, nothing returns to earth.

The SSSD was used to calculate both the LCI and LCIA results. Validated at ESA through a collaborative project in late 2018 (Wilson, 2018), the SSSD has already been used in the design of several space missions. It consists of 250 unique foreground space‐specific life cycle sustainability data sets; each contains environmental, costing, and social data, building upon Ecoinvent and ELCD E‐LCA background inventories. A process‐based methodology is used that relies on physical activity data to develop data sets derived from assessing all the known inputs of a particular process and calculating the direct impacts associated with the outputs of that process. The SSSD also includes several impact categories at the midpoint level. This is a problem‐oriented approach that quantifies and translates the life cycle impacts into themes such as climate change, ozone depletion, acidification, human toxicity, social performance, costs, and so forth. The five E‐LCA impact categories selected as part of this study relate to the five impact categories identified by ESA as being most problematic (S. M. Serrano, European Space Agency, personal communication, 18 September 2018). These are climate change, mineral resource depletion, ozone depletion, freshwater aquatic ecotoxicity, and human toxicity. Additionally, the SSSD aligns closely with a variety of widely accepted international standards and norms, which are used as the basis for this coordinated, overarching space LCSA framework (Wilson, 2019).

In terms of data collection, primary data were collected directly from the mass budget and subsystem information contained within the MÌOS engineering model that was used within the concurrent engineering process. This was then mapped to the relevant SSSD processes. For all other elements of the MÌOS life cycle that was not pertinent to data contained in the engineering model, default values contained in the SSSD as well as well‐judged estimations were used based on expert input. In particular, to fulfill MIS‐R‐03, it was assumed that the mission would be launched with the other three other missions, meaning that MÌOS was attributed a 25% share of total launch segment impacts.

For the social impacts, 31 different stakeholder groups were identified including the University of Strathclyde, ESA, ArianeGroup, plus 28 other entities. These were then matched to SSSD S‐LCA data sets that were obtained using freely available averaged national‐level data and integrated into E‐LCA data sets to represent these stakeholders based on their country of operation. This was considered appropriate since Siebert et al. (2018) state that “an organisation's conduct is highly influenced by national and regional socioeconomic conditions.” The activity variable was based on detailed working hours for each process (already contained within the SSSD). The overall social impacts were calculated as a single score for all stakeholders for the stakeholder categories of “worker” and “value chain actors (VCA).”

For LCC, a new parametric‐analogous hybrid cost model that adopts an ABC estimating approach was integrated into the SSSD E‐LCA data sets. This ABC approach treats each activity of a space mission defined by the system boundary within the ESA E‐LCA guidelines as cost pools. In particular, this mainly estimates future costs based on historical trends, with the analogous part adjusting these parametric costs for complexity, technological, and physical differences in a similar manner to Saint‐Amand and Ouziel (2015) and Ouzeil and Saint‐Amand (2015) for TRLs. The costs were calculated as a single score, taking into account relevant predefined cost categories.

**Figure 4:**
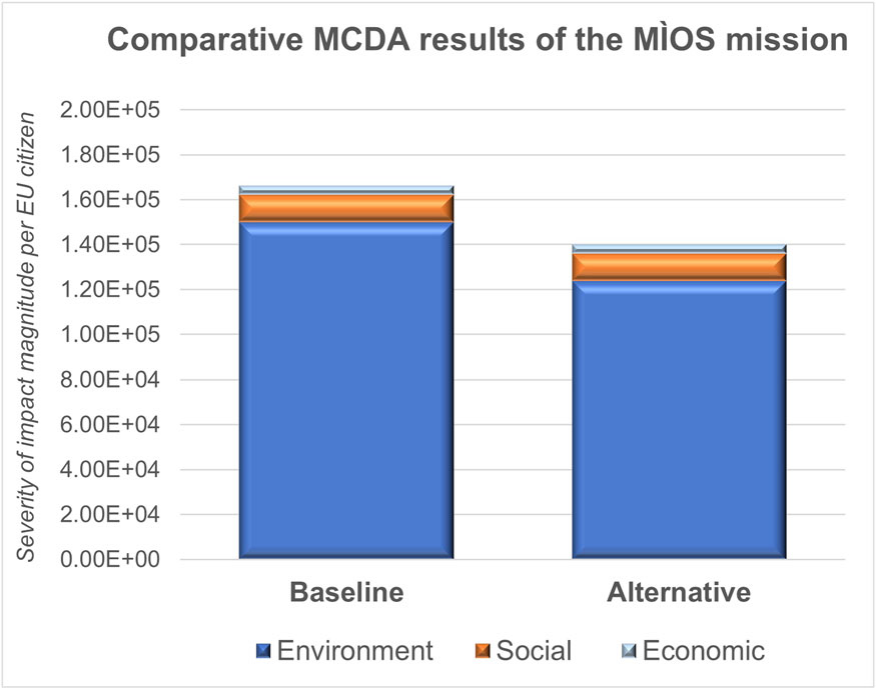
Comparative MCDA results of the MÌOS mission for the baseline and alternative design. MCDA, multicriteria decision analysis; MÌOS, Moon Ice Observation Satellite

The results of this process can be seen in Table 4. However, as can be seen, the high number of impact categories makes it difficult to determine which impact categories or sustainability dimension are most important to address. As such, the normalization and weighting factors outlined in Wilson and Vasile (2022) were used to create a single sustainability score through MCDA. This creates an “importance factor” of each sustainability pillar, relating to the severity of impact magnitude per EU citizen. More specifically, the five environmental impact categories selected for this study were normalized based on the approach recommended for the Product Environmental Footprint method (Benini et al., 2014) that related to the EU‐27 domestic inventory in 2010 per EU citizen. The normalized values were then multiplied by the Joint Research Centre recommended weighting set (Sala et al., 2018) that was reformulated to 100% based on the impact categories used. For the Social Impact category, the normalization method was based on the percentage of global companies that have not set quantitative targets linked to their societal impact for at least one key performance indicator in 2016 (PwC, 2017). This was then multiplied by the total number of active EU‐28 entities to generate a total social score for all EU entities (Eurostat, 2018). This was again multiplied by the total number of hours in one year to produce an annual social score, before being divided by the EU‐28 population in 2016 to produce the average share of total European organizational social impact per EU citizen (Eurostat, 2021). It is recognized that this approach has a very low level of robustness and work is ongoing to create a better normalization procedure. For Whole Life Cost, the normalization factor was based on the average tax rate of EU‐28 nations per citizen in 2015 to the value of the euro in the year 2000 (European Commission, 2015). The weighting factors for both Social Impact and Whole Life Cost were given a value of 100% since they each represent an individual sustainability dimension. Finally, the generated values for the environmental, social, and economic dimensions were multiplied by another weighting factor based on the relative importance of each sustainability dimension. As previously mentioned, this was based on indicators dedicated to each dimension within the 2030 Agenda for Sustainable Development (as specified by Diaz‐Sarachaga et al., 2018), which gave a split of 18% to the environmental dimension, 53% to the society dimension, and 29% to the economic dimension. Based on this approach, it was found that the final importance of impact magnitude per EU citizen for the entire sustainability score is 1.66E+05, which is composed of 1.50E+05 environmental impact, 1.22E+04 social impact, and 4.04E+03 economic impact. An overview of these results can be seen in Figure 4.

**Table 4:** LCSA results of the MÌOS mission for the baseline and alternative design

Sustainability dimension	Impact category	Unit	Method	LCIA results	
				Baseline design	Alternative design
Environment	Climate change	kg CO_2_ eq.	IPCC (Myhre et al., 2013)	1.12E+07	1.12E+07
	Freshwater aquatic ecotoxicity	PAF m^3^ day	USEtox (Fantke et al., 2002)	6.93E+07	6.92E+07
	Human toxicity	Cases	USEtox (Fantke et al., 2002)	1.88E+03	1.47E+03
	Mineral resource depletion	kg sb eq.	CML (Guinée et al., 2002)	2.58E+05	2.03E+05
	Ozone depletion	kg CFC‐11 eq.	WMO (1999)	2.17E+04	2.17E+04
Society	Social impact	Social risk	Wilson (2019)	7.70E+08	7.63E+08
Economic	Whole life cost	€ (CY:2000)	Wilson (2019)	1.19E+08	1.19E+08

Abbreviations: CML, Centrum voor Milieukunde Leiden; LCIA, Life Cycle Impact Assessment; LCSA, Life Cycle Sustainability Assessment; MÌOS, Moon Ice Observation Satellite; PAF, Potentially Affected Fraction of Species.

In terms of interpretation, the MCDA results clearly indicate that E‐LCA should be considered to be the most important sustainability dimension to address for the baseline design of the MÌOS concept. The majority of this impact came from mineral resource depletion (59.98%), which is directly attributable to the use of germanium in solar cells. A contributing factor for this result could be the fact that the Centrum voor Milieukunde Leiden “reserve base” horizon was used for this impact category (as recommended in the ESA E‐LCA guidelines), a choice that leads to germanium being particularly impactful. Other high‐scoring impact categories were human toxicity (20.18%) and ozone depletion (19.71%). The former result is largely due to the manufacturing and production of the launcher propellants and dioxins released during the production and manufacture of the germanium substrate for the solar arrays. The latter result was almost entirely due to the launch event.

In comparison, from a social perspective, it is clear that by far the largest social impact comes from the “Working Hours” stakeholder subcategory (which produces 29.30% of the total social impact). This was based on a survey conducted as part of this research at the University of Strathclyde that found that the working hours of PhD students and academics within the Department of Mechanical & Aerospace Engineering were generally longer in real terms than reported by the university. While at ESA during the ESA LCI Validation Project (Wilson, 2018), similar working patterns were also observed. This trend led to the establishment of a factor that was applied to average working times reported by each country based on OECD (Organisation for Economic Co‐operation and Development) data (OECD, 2022). As such, it was found that a very high risk factor was assigned to most countries associated with the MÌOS concept, which was the primary reason for this score. The highest VCA stakeholder subcategory was “Promoting Social Responsibility,” which produced 9.25% of the total social impact. This score was based on information contained within a report titled “Global trends in sustainability reporting,” which highlighted the low number of reporting instruments identified by country (KPMG International, 2019). In terms of how this affects the SDGs, it was found that the top five most affected SDGs represent 79.41% of the total social score, which are SDGs 8, 10, 12, 16, and 17.

Additionally, it can be seen that the majority of costs arise from labor in terms of work‐hours (6.89E+07 EUR 2000). This was closely followed by the launch segment (3.61E+07 EUR 2000), which relates almost entirely to acquisition of the Ariane 5 ECA launcher. The third most impacting cost element was transportation (9.30E+06 EUR 2000) due to the shipping of both the spacecraft and launcher components to the ESA launch site in Kourou, French Guiana, and the air travel involved for staff and/or expert participation in space mission design sessions and launch event activities.

### Alternative design

Given the MCDA results, it was considered that the germanium substrate of the solar array is the most prominent sustainability hotspot. For this reason, the first sustainable design option is to target the germanium substrate used within the solar array. The baseline design uses a triple‐junction GalnP/GaAs/Ge solar cell with a mass of 18.84 kg (including mass margins) and a conversion efficiency of 30%. The second sustainable design option is to replace hydrazine with LMP‐103S, which is an HPGP. This is because hydrazine is particularly toxic and now on the candidate list of substances to be regulated under the EU's regulation concerning the REACH regulations (European Chemicals Agency, 2022). In this regard, LMP‐103S is a flight‐proven HPGP that is marketed as being much less toxic than hydrazine and also noncarcinogenic. More specifically, LMP‐103S has a 6% higher specific impulse than hydrazine and is 24% more dense based on values based on observations from the PRISMA mission that was launched on 15 June 2010 (Dinardi, 2013). As such, it shows a 30% higher density impulse, meaning that 56.9 kg of the propellant is required in comparison to 61.2 kg of hydrazine in the MÌOS mission.

The executed sustainable design measures led to a 21.71% downsizing of the solar array and the replacement of the hydrazine propellant with LMP‐103S. This latter option was implemented since it would lead to a 7% reduction in the amount of propellant required, despite the fact that kg to kg LMP‐103S was found to perform environmentally worse than hydrazine on almost every impact category, a result confirmed by GreenSat project (Thiry et al., 2019). This impact was primarily due to ammonium dinitramide production and, in particular, the influence of nitric acid (from the production of potassium dinitramide), isopropanol, and pentane. The combination of these two sustainable design measures led to waterfall mass savings of 5.05% and a reduction in the MCDA score by 15.66% (see Figure 4). On average, the alterative design of the MÌOS concept generated average environmental savings of 8.65% across the five impact categories, a 0.91% better social performance, and a reduction of 6.62E+04 EUR 2000 in costs. The direct savings in social impact occur mainly due to the use of LMP‐103S in comparison to the risk associated with workers handling hydrazine. Additionally, as LMP‐103S is produced in Sweden and hydrazine is produced in Germany, the workers category of LMP‐103S scored significantly better for this activity, particularly relating to well‐being of staff (36.71%) and working hours (33.33%). In terms of costs, the reduction of mass meant that due to the linear nature of CERs, a cost reduction was also achieved.

Overall, it is hypothesized that the environmental savings were almost entirely due to the reduction of the solar array mass. Actually, it is suspected that the replacement of hydrazine with LMP‐103S actually suppressed the improvement measures. Proving this would be extremely challenging since tracing the full indirect impacts to a single sustainable design option is not a straightforward procedure. This is due to the interrelated nature of design decisions and the chain reaction that they can put into motion. For example, changes to the centre of mass caused by the redesign led to a reduction in the mass of the reaction wheels by 8.25%. As such, it is difficult to determine which sustainable design option primarily drove this change since both created reductions in system mass. However, despite the implementation of this sustainable design solution, it can be seen that the environment is still the driving force behind the LCSA results.

This study has demonstrated the importance of considering each sustainability pillar, even when targeting just one dimension. Moreover, the results suggest how imperative it is that system‐level technical considerations are also taken into account when designing sustainable space systems. In this regard, a space system component that performs worse environmentally, socially, and/or economically at face value may actually be the more sustainable option if it provides an optimized performance at the system level through redesign. Therefore, it can be concluded that completely replacing technologies without considering the complete system‐level performance is an inattentive and poor sustainable design choice.

Going forward, the results of this exercise can now be used to address the hotspots across the supply chain. In this regard, technological improvements can now be targeted, which include advanced manufacturing technique refinement (similar to the efforts that ESA are undertaking) (Zimdars & Izagirre, 2017) and/or the development of lightweight spacecraft materials and structures (NASA, 2012). In particular relation to the MÌOS mission, it is recommended that either the solar array size is reduced further or the germanium substrate is directly replaced, while additional efforts are made to minimize the other identified environmental hotspots within future design iterations. Additionally, while other HPGPs could be investigated for use within the MÌOS mission, switching propellants from hydrazine to LMP‐103S should not be considered as a sustainable design solution.

## EVALUATION AND DISCUSSION

Several commonalities and differences exist between the three assessment types of E‐LCA, S‐LCA, and LCC. Therefore, it is important to synthesize these in terms of the main drivers, applied methodologies, and data requirements in order to better understand the benefits and drawbacks of addressing them within a single space LCSA framework.

First, the drivers of each assessment are distinctly different. Although E‐LCA is mainly driven by environmental impact mediums, S‐LCA is primarily based on principles of social responsibility outlined within ISO 26000:2010, while LCC is commonly steered by predefined financial factors typically based on mission requirements and/or a WBS. As such, this makes the strategy and methodological choices that are determined during the goal and scope definition an extremely important element of the LCSA process to align these drivers.

In this regard, the space LCSA framework aims to follow a common goal and scope in order to reduce the effort required in impact modeling. Since current practice dictates that E‐LCA is used as the baseline methodology on which S‐LCA and LCC should be applied, there is a need to tailor these assessments to E‐LCA. In terms of S‐LCA, selecting activity variables that best accord with reference flows of processes can be extremely challenging if an organizational perspective is adopted. However, this is necessary to relate organizational social impacts to processes. This becomes even more challenging if new social indicators are created. To ensure that all indicators are quanitifed in a unified way, this places an added importance on selecting a scoring mechanism with relevancy to both quantitative and qualitative inventory data. Therefore, linking social inventory data to activity variables and then relating activity variables to quantitative references is extremely important for inventory relevancy but may limit what could be considered appropriate to reflect this relationship. In terms of LCC, the creation of costing flows is a lot simpler since they adopt a product‐based perspective like E‐LCA. However, it is important to define the cost bearer, which should generally be viewed from the perspective of the organization responsible for designing the space mission as a baseline.

In terms of data requirements, LCI data acquisition for compilation within a database may also be extremely challenging for all three assessment types, meaning that stakeholder buy‐in is particularly important for compiling an accurate and relevant LCI. Despite the varied and diverse LCI data requirements, a well‐developed sustainable design tool should look to minimize the amount of additional data that engineers are required to provide in space mission design sessions. Therefore, should dedicated environmental, social, and economic data sets be developed in accordance with the proposed space LCSA methodology, then integration of these within the same data set should sufficiently achieve this since all of the LCI data refer to a common quantitative reference. The SSSD has been created to simplify this process based on the methodological guidance offered by this framework, eliminating the need for background data collection.

The outputs of each assessment type are based on the LCIA methods outlined in the Framework Development section. Although life cycle hotspots will mostly depend on the goal and scope definition, it is hypothesized that within E‐LCA, the spacecraft, launcher, and propellant production and manufacturing will produce the greatest impact across most impact categories. In comparison, it is thought that in S‐LCA, it will mostly be affected by activities with high levels of organizational involvement (typically during spacecraft and launcher production and manufacturing), while LCC will mostly be influenced by costs from labor, launcher acquisition, and satellite operations.

Finally, it should also be noted that the impacts of each assessment are considered to be self‐contained before MCDA is applied (i.e., no direct interactions between assessment types). However, the importance of MCDA should not be understated since its ability to address the multidimensional results of LCSA is vital to the decision‐making process. Ultimately, despite the subjectivity that such an approach introduces to results (as demonstrated by Wilson & Vasile, 2022), addressing each assessment type within a single framework offers numerous benefits. Assuming that the space LCSA framework is followed and that stakeholder buy‐in can be achieved for LCI data collection, then aggregating these three assessments allows for complex environmental, social, and economic and social data to be organized in a structured and common form to generate a more comprehensive overview of life cycle sustainability impacts of space missions.

## CONCLUSION

This article outlines the potential for using LCSA as a method for designing sustainable spacecraft and presented a methodological framework for its application within the space mission design process. The framework provides a credible and compelling new method for streamlining the decision‐making process in a more systematic and coordinated fashion, with the concept of sustainable development at its core. This was demonstrated on the Phase 0/A MÌOS space mission design concept to scientifically identify and reduce adverse sustainability impacts over its entire life cycle through system redesign. As such, it is hoped that this proposed framework will contribute to the global sustainability agenda by assisting engineers to design space missions that are not only cost‐efficient, eco‐efficient, and socially responsible but also ones that can easily justify and evidence their sustainability.

### Limitations

The approach is not without its challenges. Since the solution is principally a burden‐based approach, stakeholder buy‐in may be difficult to achieve in the first instance as organizations may not want to participate in data collection or any other activity that could be seen to damage their reputation. However, shifting public perceptions may force organizations to rethink this protective stance, with 80% of citizens in EU Member States thinking that businesses and industry are not doing enough to protect the environment, according to a recent Eurobarometer survey. (European Commission, 2020).

Moreover, the use of E‐LCA, S‐LCA, and LCC indicators and normalization and/or weighting methods within the space systems design process is still not consensual, given the current lack of a standardized method and commonly agreed quantitative metrics. This particularly relates to S‐LCA due to the novelty of the approach when applied to a space system. As such, the proposed method outlined by this framework may need to be further elaborated on in the future. For this reason, an international protocol may need to be established to govern the harmonization of LCSA and/or LCE for space technologies.

Lastly, it should be noted that while the most dramatic life cycle impacts and optimization activities associated with space systems engineering are obtained in the early stages, decisions that affect the environment, society, or costs continue to be amenable to the systems approach even as the end of the system lifetime approaches. Although the presented case study of this paper was a Phase 0/A study, users of the new space LCSA framework must consider the concept's stage of design at the point of study to ensure that the most appropriate method is applied. For example, in LCC, while CERs or analogous costing is mostly applicable to early space mission concepts such as the MÌOS example, grassroots costing is more applicable to more adequately defined concepts.

### Expected outcome

The novelty of this newly proposed space LCSA framework lies in the adaptation of the current E‐LCA, S‐LCA, LCC, and MCDA methods to application to space missions. In particular, this relates to the calculation of sustainability impacts in a manner that is most relevant to industrial practice while enabling traceable decision‐making (i.e., the provision of best practice in LCSA when applied to space). This is very relevant, given the complexity of space missions as a product system.

Overall, the framework has been established as a voluntary tool with a goal of assisting industry to integrate LCSA into the design process of early space mission concepts and concurrent engineering. As such, it is not the intention of the framework to dictate which methodologies should be applied within a space LCSA study, but instead provide robust and systematic methodological guidance based on best practice. This was defined from literature reviews with reference to both the LCSA methodology and current practice within the space industry. The implementation of this framework is currently supported through the SSSD, as outlined by Wilson (2019). As such, this new space LCSA database should be seen as an extension of this framework.

Through its use, the framework can be used to illustrate how design decisions targeting a specific sustainability dimension may affect the others, since an environmentally friendly design does not necessarily mean that it is socially responsible or economically viable. In this regard, the LCSA results can be fed into the decision‐making process at either the system level (e.g., redesign activities) or the strategic level (e.g., making changes to the procurement process) to drive internal change and create a truly sustainable space sector. This can help to lower ecological burdens, avoid potential supply chain disruption, and reduce costs, all while demonstrating the interaction of each sustainability dimension.

The application of the developed space LCSA framework has already been exemplified within this article using MÌOS as a case study. However, the adoption of the technique within industry is not difficult to envisage. E‐LCA is already a requirement for all future Copernicus missions, and it is reported that it may become a mandatory element of the space mission design process at ESA in the future (Wilson & Neumann, 2022). Such a scenario would provide an ideal opportunity for LCSA to also be integrated as a complementary tool to E‐LCA (at least on an experimental basis). Not only would this help to advance the methodology but it would also ensure the widespread knowledge and/or application of the approach at all levels within the space sector.

As a result, it is expected that this approach will be used by industry for the design of next‐generation sustainable space systems, allowing conclusions to be reached based on the interactions of each sustainability dimension during the mission design process. It will therefore allow the space industry to streamline future decision‐making and monitoring in a more systematic and coordinated fashion, which accords with best practice.

## AUTHOR CONTRIBUTION

**Andrew R. Wilson**: Conceptualization; data curation; formal analysis; funding acquisition; investigation; methodology; resources; software; validation; visualization; writing—original draft. **Massimiliano Vasile**: Conceptualization; funding acquisition; project administration; supervision; writing—review and editing. **Christie Maddock**: Conceptualization; project administration; supervision; writing—review and editing. **Keith Baker**: Conceptualization; project administration; supervision; writing—review and editing.

## Data Availability

Data, associated metadata, and calculation tools are available from the corresponding author Andrew R. Wilson (andrew.r.wilson@strath.ac.uk). Requests for access to the Strathclyde Space Systems Database (SSSD) can be sent to the authors for consideration.
